# Rebuilding Stability: Exploring the Best Rehabilitation Methods for Chronic Ankle Instability

**DOI:** 10.3390/sports12100282

**Published:** 2024-10-17

**Authors:** Roberto Tedeschi, Vincenzo Ricci, Domiziano Tarantino, Luigi Tarallo, Fabio Catani, Danilo Donati

**Affiliations:** 1 Department of Biomedical and Neuromotor Sciences, Alma Mater Studiorum, University of Bologna, Via Zamboni 33, 40126 Bologna, Italy; 2 Physical and Rehabilitation Medicine Unit, Luigi Sacco University Hospital, ASST Fatebenefratelli-Sacco, 20146 Milan, Italy; 3 IRCCS Fondazione Don Carlo Gnocchi ONLUS, 50143 Florence, Italy; 4 Department of Orthopedics and Traumatology, Polyclinic of Modena, University of Modena and Reggio Emilia, 41124 Modena, Italy; 5 Physical Therapy and Rehabilitation Unit, Policlinico di Modena, 41124 Modena, Italy; 6 Clinical and Experimental Medicine PhD Program, University of Modena and Reggio Emilia, 41124 Modena, Italy

**Keywords:** chronic ankle instability, balance training, proprioception, rehabilitation, functional recovery

## Abstract

**Background:** Chronic Ankle Instability (CAI) is a common condition characterized by repeated episodes of ankle “giving way” and impaired balance, leading to functional limitations. Various rehabilitation techniques, including balance training, proprioceptive exercises, whole-body vibration (WBV), and novel approaches like stroboscopic vision, are used to address these deficits. This review evaluates the effectiveness of different rehabilitation interventions for CAI management. **Methods:** A review was conducted by analyzing 11 randomized controlled trials that investigated the impact of balance and proprioceptive training programs on CAI. The primary outcomes assessed were the Star Excursion Balance Test (SEBT), Cumberland Ankle Instability Tool (CAIT), and Foot and Ankle Ability Measure (FAAM). Methodological quality was assessed using the PEDro scale, and the risk of bias was evaluated with the ROB 2 tool. **Results:** All rehabilitation interventions demonstrated significant improvements in SEBT, CAIT, and FAAM scores. However, no single intervention was found to be consistently superior. Traditional balance training, strength exercises, BAPS, and WBV all provided meaningful functional gains. Stroboscopic vision training showed similar effectiveness compared to conventional approaches. The evidence supports a combination of balance and strength training for optimal recovery. **Conclusions:** Balance and proprioceptive exercises are effective in managing CAI, with improvements in both dynamic stability and subjective outcomes. No intervention stands out as the best, but personalized programs incorporating various methods are recommended. Future research should explore the long-term effects and potential synergies of combined interventions.

## 1. Introduction

Chronic Ankle Instability (CAI) is a complex condition involving disruptions in both sensory and motor functions [1,2,3,4]. These disruptions result in a reduced ability to process important sensory information from receptors in the foot, muscles, tendons, and ligaments, ultimately affecting the development of effective movement strategies. Recent scientific evidence suggests that Chronic Ankle Instability (CAI) is driven by a complex interplay between neurological and biomechanical factors. Neurologically, damage to mechanoreceptors within the lateral ankle ligaments significantly alters proprioceptive feedback, compromising the central nervous system’s ability to process crucial sensory information required for postural control and joint stability. This loss of sensory input results in deficits in anticipatory muscle activation and a diminished ability to generate effective motor responses during dynamic movements. Additionally, CAI is associated with neuromuscular dysfunction, characterized by delayed activation of lateral stabilizing muscles such as the peroneals, which are crucial for protecting against excessive inversion during movement. These neuromuscular deficits contribute to impaired dynamic stability, increasing the likelihood of recurrent episodes of joint instability. Biomechanically, individuals with CAI exhibit significant alterations in movement strategies. Kinematic analyses have revealed decreased dorsiflexion and increased inversion during gait and other functional activities, suggesting a restricted range of motion that further contributes to instability. These altered neuromuscular control patterns not only affect walking but also exacerbate the likelihood of injury recurrence. The integration of targeted rehabilitation interventions aimed at restoring proprioception, muscle strength, and neuromuscular control is essential to breaking this cycle of sensory and motor dysfunction. Recent studies support the efficacy of combined proprioceptive and strengthening exercise programs in improving functional stability and reducing the risk of recurrence [5,6,7,8,9,10].

CAI can manifest as either functional or mechanical instability. Functional instability typically follows an ankle sprain and is characterized by a subjective feeling of excessive mobility in the ankle, even when voluntary control over movements is maintained [11,12,13,14,15,16]. Mechanical instability, on the other hand, is associated with joint laxity, leading to movements that exceed the normal range of motion, which can be objectively measured through clinical tests [17,18,19,20]. CAI is further categorized as either primary, where no prior trauma has occurred, or secondary, which develops after one or more sprains. It is estimated that up to 70% of individuals who suffer an initial lateral ankle sprain may develop CAI in a short period. This condition is particularly common in people who participate in activities that involve running, jumping, and frequent direction changes [21,22,23,24,25]. While lateral ankle sprains (LAS) are less common in activities like dance and gymnastics, the prevalence of CAI is notably higher among individuals in these disciplines [26,27,28,29,30,31]. Currently, no standardized diagnostic criteria exist for CAI. Clinicians often assess the history of recurrent ankle sprains and any functional limitations that persist for more than a year after the first injury. The Cumberland Ankle Instability Tool (CAIT) is commonly used to measure the severity of functional deficits in individuals with CAI, with lower scores indicating greater instability [32,33,34,35,36,37,38]. Symptoms of chronic instability can develop rapidly, with recurrence occurring within 6 to 12 months of the initial trauma [39,40,41,42,43]. Physical examination typically involves assessing the mechanism of injury, which in LAS is usually linked to excessive inversion and plantar flexion. Key deficits in patients with CAI include a limited range of motion (ROM), reduced strength, altered postural control, and changes in movement strategies. Studies have shown significant impairments in single-leg balance, especially during activities like hopping and jumping, with substantial deficits also observed in dynamic postural control, often assessed using the Star Excursion Balance Test (SEBT) [44,45,46,47,48,49,50,51]. The initial injury can affect not just the ligaments but also nerve, muscle-tendon, and cartilage tissues, contributing to secondary chronic instability. Commonly associated conditions include loose bodies in the joint, impingement syndromes, osteochondral lesions, tendon injuries, and damage to the tibiofibular syndesmosis [52,53,54]. Despite the frequency of ankle sprains, many patients do not receive adequate rehabilitation [55,56], which may contribute to the development of CAI, although this relationship remains unclear. Due to the chronic nature of CAI, surgery is often considered after failed rehabilitation efforts. Around 85% of ankle sprains affect the lateral ligament complex, and most involve the anterior talofibular ligament, with the calcaneofibular ligament also frequently involved. Surgical options include anatomical reconstruction, which restores ligament tension and is often performed arthroscopically, or tenodesis, which aims to correct instability, sometimes at the cost of reduced range of motion [57,58,59]. Conservative treatments, including rehabilitation programs focused on physical exercise, have been shown to reduce the recurrence of ankle sprains and improve the management of CAI [55,60,61]. Many rehabilitation protocols combine proprioceptive and strengthening exercises, often incorporating multistation setups with up to 18 different exercises. While these programs are beneficial, their execution demands significant time and resources, and they may be less effective when performed in group settings without individualized feedback. Given the lack of consensus on the best rehabilitation approach for CAI, this review aims to explore the effectiveness of a proprioceptive exercise program in improving functional recovery in individuals with chronic ankle instability.

## 2. Methods

This review followed the JBI [62] methodology for scoping reviews. Additionally, we adhered to the PRISMA-ScR (Preferred Reporting Items for Systematic Reviews and Meta-Analyses Extension for Scoping Reviews) [63] checklist to guide the reporting process.

### 2.1. Review Question

We formulated the following research question: “*Does a proprioceptive exercise program improve functional recovery in individuals with chronic ankle instability (CAI)?*”

### 2.2. Eligibility Criteria

Studies were considered for inclusion if they fulfilled the specified criteria based on Population, Concept, and Context (PCC).

**Population (P):** The population targeted by this review included individuals diagnosed with Chronic Ankle Instability (CAI). These individuals typically had a history of at least one significant lateral ankle sprain, with recurrent episodes of instability or giving way, reported subjective feelings of ankle instability, and demonstrated objective functional deficits. Participants could include athletes, non-athletes, or physically active individuals who had experienced repeated ankle injuries leading to chronic instability. Importantly, studies focusing on acute ankle injuries or populations without a diagnosis of CAI were excluded.

**Concept (C):** The core concept examined in the review was the effectiveness of proprioceptive exercise programs in enhancing functional recovery in CAI patients. Proprioceptive exercises are designed to improve the body’s ability to sense joint position and movement, with the aim of enhancing balance, coordination, and overall functional stability. Studies were included if they evaluated the impact of isolated proprioceptive exercises or proprioceptive exercises combined with other conservative treatments (e.g., muscle strengthening). The primary outcomes of interest included improvements in dynamic balance, postural control, range of motion (ROM), and overall ankle functionality, as measured by tools such as the Star Excursion Balance Test (SEBT) and the Y Balance Test.

**Context (C):** The context of this review focused on rehabilitation settings where CAI individuals performed proprioceptive exercise programs. These could include clinical rehabilitation centers, sports facilities, or supervised home-based programs. The studies included could be conducted in various settings, such as athletic training centers, outpatient rehabilitation clinics, or research facilities. The review did not limit inclusion by geographic location, age group, or the type of healthcare system in which the interventions were delivered. However, studies were excluded if they involved surgical interventions or pharmacological treatments without a focus on exercise-based rehabilitation.

### 2.3. Exclusion Criteria

Studies that did not align with the defined PCC criteria were excluded from the review.

### 2.4. Search Strategy

A thorough search strategy was implemented to identify studies relevant to this systematic review. Several databases were searched to ensure a comprehensive examination of the existing literature. The databases consulted, the search terms utilized, and the search timeframe are outlined below.

Databases Used for Search:

PubMed

Cochrane Library (CENTRAL)

PEDro (Physiotherapy Evidence Database)

Scopus

Web of Science

Search Period: The literature search was conducted between April 2024 and August 2024, with a final update in August 2024 to include the latest relevant studies.

Search Strategy: A combination of Medical Subject Headings (MeSH) and relevant keywords was applied to cover the population, intervention, and outcomes of interest. Boolean operators (AND, OR) were used to combine search terms efficiently and ensure comprehensive coverage.

- **PubMed Search String:** ((“Chronic Ankle Instability” [Title/Abstract]) OR (“Ankle Instability” [Title/Abstract]) OR (“Joint Instability” [MeSH Terms]) AND (“Ankle” [MeSH Terms])) AND ((“Proprioception” [MeSH Terms]) OR (“Proprioceptive Exercise” [Title/Abstract]) OR (“Sensorimotor Training” [Title/Abstract]) OR (“Neuromuscular Training” [Title/Abstract]) OR (“Rehabilitation” [MeSH Terms])) AND (“Randomized Controlled Trial” [Publication Type])
- **Cochrane Library CENTRAL:**
  #1 “Chronic Ankle Instability”
  #2 “Ankle Instability”
  #3 “Joint Instability” AND “Ankle”
  #4 #1 OR #2 OR #3
  #5 “Proprioception” OR “Proprioceptive Exercise” OR “Sensorimotor Training” OR “Neuromuscular Training”
  #6 #4 AND #5
- **PEDro Search String:** “Chronic Ankle Instability” OR “Ankle Instability” AND “Proprioception” OR “Proprioceptive Exercise” OR “Balance Training” OR “Sensorimotor Training”
- **Scopus:** (TITLE-ABS-KEY (“Chronic Ankle Instability”) OR TITLE-ABS-KEY (“Ankle Instability”) OR TITLE-ABS-KEY (“Joint Instability”))
  AND (TITLE-ABS-KEY (“Proprioception”) OR TITLE-ABS-KEY (“Proprioceptive Exercise”) OR TITLE-ABS-KEY (“Sensorimotor Training”) OR TITLE-ABS-KEY(“Neuromuscular Training”))
  AND (TITLE-ABS-KEY (“Randomized Controlled Trial”) OR TITLE-ABS-KEY (“Rehabilitation”))
- **Web of Science**: (“Chronic Ankle Instability” OR “Ankle Instability” OR “Joint Instability”) AND TOPIC: (“Proprioception” OR “Proprioceptive Exercise” OR “Balance Training” OR “Sensorimotor Training” OR “Neuromuscular Training” OR “Rehabilitation”) AND TOPIC: (“Randomized Controlled Trial”)

### 2.5. Search Process

The search across these databases yielded a total of 663 articles. After removing duplicates, 214 unique articles remained. These were screened for relevance based on titles and abstracts, leading to the exclusion of 203 articles. The remaining 11 articles were assessed in full text against the eligibility criteria, resulting in the exclusion of five studies that did not meet the criteria. Ultimately, eight studies were included in the final review. To enhance the transparency of the review process, we further clarified the exclusion criteria. Studies were excluded if they had a sample size considered too small to provide adequate statistical power, generally fewer than 15 participants per group, which could compromise the generalizability of the results. Additionally, studies that lacked proper controls or randomization procedures or that did not include relevant outcome measures such as balance, proprioception, or functional recovery were excluded. Studies with high risk of bias, particularly those without blinding or those that had inadequate follow-up periods, were also removed from consideration. This step ensured that only high-quality randomized controlled trials (RCTs) were included, allowing for a more rigorous evaluation of the interventions.

### 2.6. Study Selection

The study selection process followed an approach tailored for a scoping review. The initial search results were compiled and organized using Zotero, where duplicates were removed. Screening was carried out in two phases: first, a review of titles and abstracts, followed by a full-text assessment. Both phases were conducted independently by two reviewers, with any disagreements resolved by a third reviewer. The selection process adhered to the PRISMA 2020 guidelines [64], ensuring a transparent and reliable method. This rigorous approach was designed to comprehensively identify articles relevant to the research question while maintaining a systematic and thorough review process.

### 2.7. Data Extraction and Data Synthesis

Data extraction for the scoping review was carried out using a form modeled on the JBI tool, capturing essential information such as author names, publication country and year, study design, patient demographics, outcomes, interventions, procedures, and other relevant factors. Descriptive analyses were performed on the extracted data, and the findings were numerically presented to illustrate the distribution of studies. The review process was clearly documented to ensure transparency, with data summarized in tables for straightforward comparison and analysis of the key features and results of the studies.

## 3. Results

**Figure 1:**
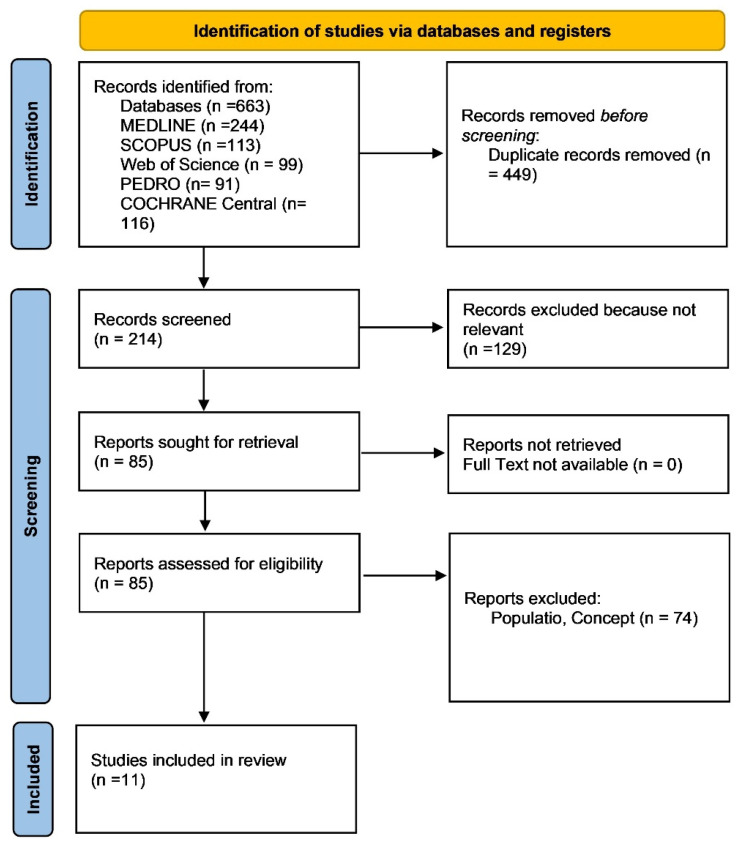
Preferred reporting items for systematic reviews and meta-analyses 2020 (PRISMA) flow diagram.

As shown in the PRISMA 2020 flow diagram (Figure 1), out of the 113 records initially identified through the literature search, 105 were excluded, leaving 8 articles for inclusion (Table 1). The quality of the included studies was evaluated using the PEDro scale and the RoB-2 tool, which assessed their methodological rigor and risk of bias (Table 2).

Although the PEDro scale and RoB-2 tool were utilized to assess the methodological quality of the included studies, a more detailed discussion of how certain biases may have impacted the overall findings is warranted. Specifically, the lack of proper blinding in participants and assessors in several studies is a notable source of performance and detection bias. In cases where participants were not adequately blinded to their group assignment, this could have influenced their behavior during the intervention, potentially leading to an overestimation of the perceived effectiveness of the treatments. Similarly, the absence of blinding among assessors may have resulted in biased outcome measurements, particularly in subjective measures such as self-reported functional ability. Furthermore, some studies featured small sample sizes and short follow-up periods, which limited the statistical power and generalizability of the results, potentially introducing attrition bias. These methodological shortcomings could have contributed to inconsistencies in outcomes across studies, especially when evaluating the long-term effects of the various rehabilitation strategies. By providing a more thorough analysis of the specific biases present in individual studies, this review enhances the robustness of the evaluation of the overall effectiveness of the interventions, thereby increasing the transparency of the review process and the validity of its conclusions.

Table 1 summarizes the control groups’ interventions, ensuring clarity on how they were designed in each study. In several studies, the control group was either given no intervention or engaged in typical physical activities without specific rehabilitation exercises. In some cases, participants continued their usual training regimen, providing a baseline for comparing the effects of the experimental interventions. This distinction is crucial for understanding the relative efficacy of rehabilitation programs against natural recovery or maintenance conditions.

### 3.1. Star Excursion Balance Test (SEBT)

The SEBT was one of the most common outcomes used across the studies to measure dynamic balance. The SEBT assesses reach distances in various directions (anterior, posteromedial, posterolateral, etc.) and is a well-established measure for balance and functional stability.

- **Four-Week Ankle-Rehabilitation Programs for Adolescent Athletes (M. Spencer Cain, 2020)** [26]: All three intervention groups (Elastic Band, BAPS, Combined) showed significant improvements in SEBT reach compared to the control group. However, there was no statistically significant difference between the intervention groups, indicating that all approaches had a similar effect on improving dynamic balance in adolescents with CAI.
- **Short-Term Effects of Balance Training with Stroboscopic Vision (Kyung-Min Kim, 2021)** [43]: Both the balance training and stroboscopic vision groups significantly improved SEBT scores compared to the control. There was no significant difference between the two intervention groups, suggesting that both methods were equally effective in enhancing balance.
- **Effects of 6 Weeks of Balance Training (D. Cruz-Diaz, 2015)** [44]: Athletes in the balance training group showed significant improvements in SEBT scores in the anterior, posteromedial, and posterolateral directions compared to the control group (*p* = 0.001). The control group, which did only the usual training, did not show comparable improvements, indicating the benefit of balance training in enhancing dynamic stability.
- **Effects of a 4-Week BAPS Protocol (Mary Spencer Cain, 2015)** [42]: Participants in the BAPS intervention group improved their SEBT scores significantly compared to the control group. The use of the Biomechanical Ankle Platform System (BAPS) led to notable improvements in balance, especially in high school athletes with CAI.
- **Balance- and Strength-Training Protocols (Emily A. Hal, 2018)** [45]: Both the balance and strength-training groups showed significant improvements in SEBT scores compared to the control. Although the balance training group demonstrated greater improvements, the difference between the balance and strength groups was not statistically significant.
- **Wobble Board Rehabilitation (Shelley W. Linens, 2016)** [46]: The Wobble Board intervention significantly improved SEBT scores compared to the control group, demonstrating its effectiveness in enhancing dynamic balance in individuals with CAI.
- **Whole-body Vibration and Balance Training (Rafael Sierra-Guzmán, 2018)** [47]: Participants in both the WBV (whole-body bibration) and non-vibration (NVB) groups improved SEBT scores, but the WBV group showed greater improvements in the short term. However, long-term benefits seemed to diminish, especially in the WBV group.
- **Whole-body Vibration and Balance Training on Female Athletes (Wen-Dien Chang, 2021)** [48]: The WBV group showed significant improvements in the anteromedial, posterolateral, and lateral directions of the SEBT compared to the control group. The balance training group, however, improved in all SEBT directions, suggesting that balance training without vibration was more effective in some aspects.
- **Comparative Effects of Neuromuscular and Strength-Training Protocols (Kyung-Min Kim, 2022)** [51]: Both the neuromuscular and strength-training groups significantly improved SEBT scores compared to the control group. The neuromuscular training group showed more marked improvements, though these were not significantly better than those seen in the strength-training group.

### 3.2. Cumberland Ankle Instability Tool (CAIT)

The CAIT measures subjective feelings of ankle instability, with lower scores indicating greater instability.

- **Four-Week Ankle-Rehabilitation Programs for Adolescent Athletes (M. Spencer Cain, 2020)** [26]: All rehabilitation groups (Elastic Band, BAPS, Combined) reported significant improvements in CAIT scores, indicating that participants felt less unstable after the interventions. However, there was no statistically significant difference between the groups.
- **Short-Term Effects of Balance Training with Stroboscopic Vision (Kyung-Min Kim, 2021)** [43]: Both the balance and stroboscopic vision training groups reported significant improvements in CAIT scores, indicating reduced subjective feelings of ankle instability compared to the control. No significant difference was noted between the two intervention groups.
- **Effects of 6 Weeks of Balance Training (D. Cruz-Diaz, 2015)** [44]: The balance training group significantly improved CAIT scores compared to the control, indicating that participants in the intervention group felt less unstable after six weeks of training.
- **Comparative Effects of Neuromuscular and Strength-Training Protocols (Kyung-Min Kim, 2022)** [51]: Both the neuromuscular and strength-training groups improved CAIT scores significantly compared to the control. Participants reported feeling more stable after 8 weeks of training.

### 3.3. Foot and Ankle Ability Measure (FAAM)

The FAAM is a subjective measure of functional ability in individuals with ankle instability, with higher scores indicating better function.

- **Four-Week Ankle-Rehabilitation Programs for Adolescent Athletes (M. Spencer Cain, 2020)** [26]: All three rehabilitation groups showed significant improvements in FAAM scores, suggesting that all the interventions positively impacted the participants’ perceived functional ability. However, no statistically significant difference was found between the groups.
- **Short-Term Effects of Balance Training with Stroboscopic Vision (Kyung-Min Kim, 2021)** [43]: Both the balance and stroboscopic training groups significantly improved FAAM scores compared to the control group. Again, no significant difference was found between the intervention groups.
- **Comparative Effects of Neuromuscular and Strength-Training Protocols (Kyung-Min Kim, 2022)** [51]: Both neuromuscular and strength-training interventions significantly improved FAAM scores compared to the control. Functional outcomes improved similarly across both intervention groups.

### 3.4. Time-in-Balance Test

The Time-in-Balance Test measures how long a participant can maintain balance on an unstable surface.

- **Four-Week Ankle-Rehabilitation Programs for Adolescent Athletes (M. Spencer Cain, 2020)** [26]: The Time-in-Balance Test showed significant improvements in all three rehabilitation groups compared to the control, but no notable difference was found between the intervention groups.
- **Effects of a 4-Week BAPS Protocol (Mary Spencer Cain, 2015)** [42]: The BAPS group showed significant improvements in the Time-in-Balance Test compared to the control, demonstrating the effectiveness of the BAPS system in improving static and dynamic balance.

### 3.5. Side-Hop Test

The Side-Hop Test evaluates dynamic stability and agility by measuring how quickly participants can hop from side to side.

- **Four-Week Ankle-Rehabilitation Programs for Adolescent Athletes (M. Spencer Cain, 2020)** [26]: All three intervention groups improved significantly in the Side-Hop Test compared to the control, indicating enhanced agility and dynamic stability. No differences between intervention groups were statistically significant.
- **Effects of a 4-Week BAPS Protocol (Mary Spencer Cain, 2015)** [42]: The BAPS intervention improved Side-Hop Test performance significantly compared to the control group, showing enhanced dynamic balance and stability.

### 3.6. Foot-Lift Test

The Foot-Lift Test assesses postural control by counting the number of foot-lifts during a balance task.

- **Four-Week Ankle-Rehabilitation Programs for Adolescent Athletes (M. Spencer Cain, 2020)** [26]: The Foot-Lift Test showed improvements in all three intervention groups, indicating better postural control compared to the control group.
- **Effects of a 4-Week BAPS Protocol (Mary Spencer Cain, 2015)** [42]: The BAPS group showed significant improvements in the Foot-Lift Test, further supporting the use of this system to improve balance and postural control in athletes with CAI.

### 3.7. Numeric Rating Scale (NRS)

The NRS is used to measure pain intensity, with lower scores indicating less pain.

- **Effects of 6 Weeks of Balance Training (D. Cruz-Diaz, 2015)** [44]: Participants in the balance training group reported significantly reduced pain on the NRS compared to the control group (*p* < 0.05). This suggests that balance training also helped alleviate pain in athletes with CAI.

### 3.8. Figure-8 Hop Test

This test measures agility and the ability to change direction while maintaining stability.

- **Four-Week Ankle-Rehabilitation Programs for Adolescent Athletes (M. Spencer Cain, 2020)** [26]: Significant improvements were observed in all intervention groups compared to the control. However, no significant differences were noted between the three rehabilitation programs.
- **Effects of a 4-Week BAPS Protocol (Mary Spencer Cain, 2015)** [42]: The BAPS group showed improvements in the Figure-8 Hop Test compared to the control group, indicating enhanced agility and stability.

### 3.9. Balance Error Scoring System (BESS)

The BESS test evaluates postural stability by counting errors during a balance task.

- **Balance- and Strength-Training Protocols (Emily A. Hal, 2018)** [45]: Both the balance and strength-training groups showed significant improvements in BESS scores compared to the control. The balance training group performed better, but the difference was not statistically significant.

## 4. Discussion

The purpose of this review was to evaluate the effectiveness of various rehabilitation interventions aimed at improving balance and functional recovery in individuals with Chronic Ankle Instability (CAI). The analysis included different training programs such as balance and proprioceptive exercises, whole-body vibration (WBV), the Biomechanical Ankle Platform System (BAPS), and innovative methods like stroboscopic vision training. Across all studies, the interventions consistently showed improvements in balance and functional outcomes, with measures like the Star Excursion Balance Test (SEBT), the Cumberland Ankle Instability Tool (CAIT), and the Foot and Ankle Ability Measure (FAAM) being central to assessing these effects.

The SEBT was the most widely used measure of dynamic balance in the studies and proved to be a valuable tool for evaluating the effectiveness of rehabilitation in CAI patients. Almost every intervention led to significant improvements in SEBT performance, particularly in the anterior, posteromedial, and posterolateral directions—critical movements that reflect real-world functional demands. These improvements suggest that balance-focused training, regardless of the specific method used, can successfully enhance postural control and dynamic stability in individuals with CAI. This is particularly relevant since dynamic balance deficits are a key issue for those suffering from CAI, as the condition often leads to repeated episodes of ankle instability during complex movements.

However, what emerged from the evidence is that no single intervention was consistently superior to others. While it is clear that no single intervention consistently outperformed others, it is essential to explore potential factors that may have contributed to the variability in outcomes across studies. Differences in participant characteristics, such as age, activity level, or the severity of Chronic Ankle Instability (CAI), may have influenced the effectiveness of the interventions. For example, younger individuals or athletes might experience greater neuromuscular adaptation and thus respond more favorably to proprioceptive and balance training, whereas older adults or those with long-standing CAI might require longer or more intensive interventions to achieve similar results. Additionally, individuals with mild CAI may benefit more from conservative rehabilitation approaches, while those with more severe mechanical instability might require combined or more advanced interventions.

For example, in Spencer Cain’s 2020 study [26], no statistically significant difference was observed between the elastic band, BAPS, or combined intervention groups, although all showed notable improvements in SEBT performance. This suggests that while these rehabilitation techniques are effective, they may not differ dramatically in their impact on balance improvements. Similarly, stroboscopic vision training, which challenges the sensorimotor system by restricting visual input, showed promising results in enhancing SEBT scores in Kyung-Min Kim’s 2021 study [43] but did not outperform traditional balance training. This points to the possibility that more innovative approaches like stroboscopic vision may not offer substantial advantages over conventional methods, although they may still serve as valuable tools for engaging specific patient populations, such as athletes who require heightened sensory awareness. The combination of balance and proprioceptive training is strongly supported by theories of motor control and neuroplasticity. Motor control theories emphasize that repeated practice of balance and proprioceptive tasks can improve neuromuscular coordination by enhancing the body’s ability to anticipate and respond to destabilizing forces. This is especially relevant for individuals with Chronic Ankle Instability (CAI), where the neuromuscular system is often deficient in its ability to quickly adapt to perturbations. Neuroplasticity, the capacity of the nervous system to reorganize itself in response to training, plays a crucial role in rehabilitation. For example, interventions like stroboscopic vision training challenge the visual and proprioceptive systems, promoting sensory reweighting. This process enables the body to rely more on proprioceptive feedback when visual input is limited, leading to better integration of sensory information and improved functional stability. These principles suggest that balance and proprioceptive interventions targeting neuroplastic changes can stimulate neural adaptations in the sensorimotor system, improving dynamic stability and overall functional recovery in CAI patients [66,67,68]. The use of novel interventions such as stroboscopic vision and whole-body vibration (WBV) is grounded in their ability to challenge the sensory systems in ways that enhance neuromuscular control and proprioception. Stroboscopic vision training reduces the availability of visual input, forcing the body to rely more heavily on proprioceptive feedback and internal cues for postural control and stability. This mechanism, known as sensory reweighting, may enhance proprioceptive sensitivity by promoting adaptations in how the brain processes sensory information. In patients with Chronic Ankle Instability (CAI), this could result in improved joint awareness and dynamic stability when visual input is not fully available, such as during rapid movements or complex sports activities. Whole-body vibration (WBV), on the other hand, stimulates the mechanoreceptors in the muscles, tendons, and joints, potentially enhancing neuromuscular activation and coordination. The mechanical oscillations produced by WBV create small, rapid perturbations that the neuromuscular system must counteract to maintain stability, leading to improved proprioceptive control and muscle response. These adaptations could be particularly beneficial for individuals with CAI, as they often experience deficits in neuromuscular activation and delayed muscle responses during functional tasks [69,70,71].

The subjective outcomes, assessed through tools like the CAIT and FAAM, mirrored the objective improvements seen with the SEBT. Patients across multiple studies reported feeling more stable and functional after completing their respective rehabilitation programs. The consistency of improvements in CAIT scores, seen in studies such as those by Cruz-Diaz (2015) [44] and Kyung-Min Kim (2022) [51], reinforces the idea that proprioceptive and neuromuscular training directly address the underlying deficits in CAI. Patients frequently report a sense of their ankle “giving way,” and the reduction in these sensations post-intervention reflects not only improved physical stability but also increased confidence in the joint’s functionality. This subjective improvement is critical, as individuals with CAI often avoid certain movements or activities due to fear of re-injury, which in turn can lead to a decrease in overall physical activity and quality of life.

The evidence also supports the role of strength training, though it appears to be most effective when combined with other forms of rehabilitation. For instance, Emily A. Hal’s 2018 study [45] found that while strength training led to functional improvements, balance-focused interventions had a slightly greater impact on dynamic stability, as measured by the SEBT. This suggests that strength training alone may not be sufficient to address the complex sensorimotor deficits present in CAI. Strengthening exercises, such as those using elastic bands, are valuable for reinforcing muscle support around the ankle joint, but they may not be enough to improve the intricate balance and proprioception needed for functional recovery in CAI. Thus, incorporating strength training as a complementary element rather than the core focus appears to yield better overall results.

Whole-body vibration (WBV) training is another method that generated mixed results. In the studies by Sierra-Guzmán (2018) and Wen-Dien Chang (2021) [48], WBV showed significant short-term improvements in SEBT scores, particularly in the anteromedial and posterolateral directions. However, its long-term efficacy was less clear, with some studies indicating that the benefits of WBV diminished over time. This raises questions about whether WBV provides enough sustained challenge to the sensorimotor system to promote lasting improvements. While it may offer a useful boost in the early stages of rehabilitation, its role as a long-term intervention remains uncertain. Traditional balance training, without the added complexity of vibration, often produced comparable or even superior results, particularly in terms of sustained improvements in functional outcomes.

In contrast, the BAPS system, which provides a progressive challenge to balance by increasing the instability of the platform, was found to be effective but not remarkably better than simpler interventions like elastic band exercises or wobble board training. The 2015 study by Mary Spencer Cain [42] showed that while the BAPS system improved SEBT and other balance measures, it did not significantly outperform other methods. This suggests that while the BAPS system can be a useful tool in structured rehabilitation programs, it may not offer unique advantages over more accessible and widely used balance training techniques. The structured progression of instability offered by the BAPS may be beneficial for certain patients, especially those requiring graded challenges, but its overall effectiveness seems to align with more traditional approaches.

Several limitations need to be considered when interpreting the results of these studies. Firstly, the heterogeneity of the interventions makes direct comparisons difficult. Although all the studies focused on improving balance and proprioception, the specific exercises, intensity, and duration of the programs varied, which could influence the outcomes. Furthermore, the follow-up periods in most studies were relatively short, often limited to immediate post-intervention assessments, which raises concerns about the long-term sustainability of the improvements seen. For instance, the short-term gains observed with WBV may not translate into long-term functional recovery, as indicated by the diminished effects over time in some studies. Additionally, blinding and randomization were not always clearly reported, as in the case of Cruz-Diaz (2015) [44] and Hal (2018) [45], potentially introducing bias in outcome assessments. Finally, many studies had relatively small sample sizes, limiting the generalizability of their findings.

Despite these limitations, the review provides valuable insights for clinical practice. The evidence strongly supports the use of balance and proprioceptive training as central components of CAI rehabilitation. While no single method was definitively superior, the consistency of improvements across interventions suggests that clinicians have flexibility in tailoring rehabilitation programs to individual patients’ needs and available resources. This flexibility is crucial, given that patients with CAI may present with different levels of severity, functional goals, and activity demands. Personalizing rehabilitation by combining various methods, such as incorporating both strength and balance exercises, may yield the best outcomes, particularly for athletes or highly active individuals who require both stability and strength in dynamic movements. Although the studies did not identify a single superior method across all populations, certain tendencies emerged when comparing subgroups. For example, athletes—especially adolescent and high-performance individuals—tended to show more significant improvement in dynamic stability measures, such as the SEBT, when engaging in proprioceptive and balance training programs. This finding aligns with similar meta-analyses in populations recovering from knee surgery or lower leg bone fractures, where early and intensive proprioceptive training yielded better functional outcomes in physically active individuals. Conversely, younger or less physically active populations, such as adolescents, appeared to benefit more from simpler balance and strength exercises, similar to rehabilitation protocols used in non-athletic injury recovery. These trends suggest that tailored rehabilitation approaches may be necessary to maximize recovery depending on the patient’s activity level and age group.

This review demonstrates that a variety of rehabilitation techniques, from traditional balance training to innovative methods like stroboscopic vision and WBV, are effective in improving both objective and subjective outcomes in patients with CAI. The evidence suggests that a combination of approaches tailored to the specific needs of the individual may offer the best results in terms of functional recovery and sustained stability. Future research should focus on exploring the long-term effectiveness of these interventions and the potential benefits of combining multiple rehabilitation strategies to optimize outcomes for individuals with CAI.

### Implications for Clinical Practice

In clinical practice, balance and proprioceptive exercises should be central to rehabilitation programs for individuals with CAI, given their consistent effectiveness in improving both balance and functional stability. Clinicians can confidently use a variety of interventions, such as elastic bands, wobble boards, and BAPS, as they yield comparable results. The key is to personalize rehabilitation based on the patient’s activity level and specific needs, potentially integrating strength exercises to complement balance training. Short-term tools like WBV may offer an initial boost but should be supplemented with long-term stability exercises. Ultimately, flexible, tailored programs that address both proprioceptive deficits and functional demands will provide the most beneficial outcomes for CAI patients. To address the gaps in current research, future studies should focus on robust designs that can provide more definitive evidence regarding the long-term effects of CAI rehabilitation. Longitudinal studies that follow patients over extended periods would be particularly valuable, as they can capture the sustainability of functional improvements and the recurrence of instability. Additionally, multicenter randomized controlled trials (RCTs) involving diverse populations would enhance the generalizability of the findings, allowing for a broader understanding of how different interventions work across various demographics and activity levels. Furthermore, future research should integrate objective biomechanical measures such as kinematic analysis and electromyography (EMG). These tools can provide insights into the neuromuscular adaptations that occur as a result of different rehabilitation protocols. For example, kinematic data could help quantify improvements in joint range of motion, while EMG can assess muscle activation patterns and their changes over time. Including these objective measures alongside clinical outcomes would allow for a more comprehensive evaluation of the efficacy of CAI rehabilitation interventions and help identify the mechanisms behind functional recovery [72,73].

Key Points:

**Balance and proprioceptive training** are essential for improving stability and function in CAI patients.

**No single intervention is superior**—clinicians can choose from elastic bands, wobble boards, or BAPS based on patient needs.

**Personalized rehabilitation** should integrate both balance and strength training for optimal results.

**Whole-body vibration (WBV)** can provide short-term benefits but should be supplemented with other exercises for long-term stability.

**Tailoring programs** to the patient’s activity level and specific functional goals will maximize recovery outcomes.

## 5. Conclusions

Balance and proprioceptive training are effective for improving both objective and subjective outcomes in individuals with CAI. While no single intervention stands out as superior, all approaches, including traditional balance exercises, strength training, and innovative methods like WBV and stroboscopic vision, offer meaningful improvements. Personalizing rehabilitation programs based on patient needs and integrating a combination of these methods will lead to the best functional recovery. A step-by-step approach is suggested to help clinicians integrate multiple rehabilitation techniques for Chronic Ankle Instability (CAI), tailored to patient characteristics such as age, activity level, and sport type. **Initial Assessment**: Evaluate CAI severity, identify deficits in proprioception, and consider patient-specific needs. **Early-Phase Rehabilitation**: Begin with basic balance and proprioceptive exercises, introducing stroboscopic vision training for athletes. **Strength and Neuromuscular Training**: Incorporate resistance exercises and whole-body vibration (WBV) to target key muscles. **Advanced Functional Training**: Progress to sport-specific drills, using biomechanical tools like electromyography (EMG) to assess improvements. **Maintenance and Return to Play**: Develop a long-term maintenance program and clear return-to-play criteria based on functional recovery. Future research should focus on long-term effectiveness and the potential benefits of combined rehabilitation strategies.

## Figures and Tables

**Table 1:** Summary of Studies on Rehabilitation Interventions for Chronic Ankle Instability (CAI).

Title	Author, Year	Sample Size (I/C)	Population	Experimental Intervention Group	Control Group	Outcome (Measures)	Outcome Assessment	Results
Four-Week Ankle-Rehabilitation Programs in Adolescent Athletes with Chronic Ankle Instability	M. Spencer Cain, 2020 [26]	43 (BAPS = 10, Elastic band = 12, Combined = 10, Control = 11)	CAI, adolescents	BAPS group: 5 rotations (clockwise and counterclockwise) for 40 s each, changing direction every 10 s, 3 sessions per week for 4 weeks	Elastic band group: 3 sets of 10 repetitions of plantar flexion, dorsiflexion, inversion, and eversion. The combined group performed both elastic band and BAPS programs. The control group received no intervention.	SEBT, Time-in-balance test, Foot-lift test, Side-hop test, Figure-8 hop test, FAAM, CAIT	3 days after the 4-week intervention	All three rehabilitation groups showed improved balance and function compared to the control but no statistically significant differences between the rehabilitation groups.
Short-Term Effects of Balance Training with Stroboscopic Vision for Patients with Chronic Ankle Instability: A Single-Blinded Randomized Controlled Trial	Kyung-Min Kim, 2021 [43]	78 (Balance training = 26, Stroboscopic training = 26, Control = 26)	CAI	Balance training group: multimodal exercises supervised, addressing static and dynamic balance tasks	Stroboscopic + Balance training; Control group received no intervention	SEBT, Ankle dorsiflexion range of motion, Self-reported instability, and Ankle functional status	Week 6, at the end of the program	Both balance and stroboscopic training groups significantly improved SEBT compared to the control. No statistically significant difference between balance and stroboscopic groups.
Effects of 6 Weeks of Balance Training on Chronic Ankle Instability in Athletes: A Randomized Controlled Trial	D. Cruz-Diaz, 2015 [44]	70 (35/35)	CAI, athletes	Balance training with 7 different activities, 3 sessions per week for 6 weeks	Control group followed regular lower limb strength training only	SEBT, CAIT, NRS	Week 6, at the end of the program	SEBT showed significant improvements in the anterior, posteromedial, and posterolateral directions in the intervention group.
Effects of a 4-Week Biomechanical Ankle Platform System Protocol on Balance in High School Athletes With Chronic Ankle Instability	Mary Spencer Cain, 2015 [42]	22 (11/11)	CAI, athletes	BAPS group: 3 sessions per week for 4 weeks	Control group received no intervention	SEBT, Time-in-balance test, Foot-lift test, Side-hop test	Week 4, at the end of the program	BAPS rehabilitation significantly improved balance in high school athletes with CAI.
Balance- and Strength-Training Protocols to Improve Chronic Ankle Instability Deficits, Part I: Assessing Clinical Outcome Measures	Emily A. Hal, 2018 [45]	47 (Balance = 17, Elastic band = 16, Control = 14)	CAI	Balance training: single-leg balance exercises, 20-min sessions, 3 times per week for 6 weeks	Strength training: elastic band exercises for dorsiflexion, inversion, and eversion; plantar flexion on a step; PNF for slow inversion. Control group cycled for 20 min at moderate intensity.	SEBT, Isokinetic strength testing, BESS	Week 6, at the end of the program	Both balance and strength groups showed significant improvements in SEBT. The balance group showed a greater effect, but the difference was not statistically significant.
Wobble Board Rehabilitation for Improving Balance in Ankles with Chronic Instability	Shelley W. Linens, 2016 [46]	34 (17/17)	CAI	Wobble board group: 5 rotations (clockwise and counterclockwise) for 40 s each, 3 sessions per week for 4 weeks	Control group received no intervention	SEBT, Foot-lift test, Time-in-balance test, Side-hop test, Figure-8 hop test	Week 4, at the end of the program	Wobble board training effectively improved functional recovery and dynamic balance, though not consistently across all outcomes.
Whole-body Vibration Training and Balance in Recreational Athletes with Chronic Ankle Instability	Rafael Sierra-Guzmán, 2018 [47]	51 (WBV = 17, NWBV = 17, Control = 17)	CAI, recreational athletes	BOSU balance training	BOSU + vibration; Control group received no intervention	SEBT, Biodex Balance System	48 h after the last session and 6 weeks post-intervention	The only significant difference between VIB and NWBV groups was seen between post-training assessments. Long-term effects seemed diminished, particularly in the vibration group.
Effects of Whole-body Vibration and Balance Training on Female Athletes with Chronic Ankle Instability	Wen-Dien Chang, 2021 [48]	63 (WBV = 21, Balance = 21, Control = 21)	CAI, female athletes	Balance training group: exercises performed using BOSU	Balance training + whole-body vibration; Control group received no intervention	SEBT	Week 6, at the end of the program	WBV group showed significant improvements in SEBT in the anteromedial, posterolateral, and lateral directions compared to the control. The balance training group showed significant improvements in all SEBT directions.
Balance Training Improves Function and Postural Control in Those with Chronic Ankle Instability	Patrick O. Mckeon, 2008 [49]	31 (Balance = 16, Control = 15)	CAI, adolescents	Balance training group: single-leg exercises with increasing difficulty, 3 sessions per week for 4 weeks	Control group maintained their normal activity levels	SEBT, Foot and Ankle Disability Index, Center of pressure (COP), Time-to-boundary (TTB)	Week 4, at the end of the program	Balance training improved postural control, dynamic stabilization during single-leg stance, and functional recovery, with SEBT improvements seen primarily in the posteromedial and posterolateral directions.
Comparative Effects of Neuromuscular and Strength-Training Protocols on Pathomechanical, Sensory-Perceptual, and Motor-Behavioral Impairments in Patients with Chronic Ankle Instability: Randomized Controlled Trial	Kyung-Min Kim, 2020 [51]	72 (Neuromuscular = 24, Strength = 24, Control = 24)	CAI, athletes	Neuromuscular training: 6 exercises with progressive difficulty over 16 sessions (8 weeks)	Strength training: elastic band exercises for inversion, eversion, dorsiflexion, and plantar flexion. Control group received no intervention.	SEBT, CAIT, FAAM, FAAM-Sport, Ankle dorsiflexion range of motion (WBLT)	Week 8, at the end of the program	Both neuromuscular and strength training groups showed significant improvements in all outcomes, with no significant differences between the two groups.
A Randomized Controlled Trial Comparing Rehabilitation Efficacy in Chronic Ankle Instability	Cynthia J., 2016 [65]	40 (20/20)	CAI	Wobble board group: 5 rotations (clockwise and counterclockwise) for 40 s each, 3 sessions per week for 4 weeks	Elastic band training: 4 directions (plantar flexion, dorsiflexion, inversion, eversion)	SEBT, CAIT, FAAM, Foot-lift test, Time-in-balance, Figure-of-8 hop, Side-hop test	1–3 days after the 4-week program	All clinical tests improved post-intervention in both groups, with no statistically significant performance differences between groups.

Legend: BAPS: Biomechanical Ankle Platform System, BESS: Balance Error Scoring System, CAI: Chronic Ankle Instability, CAIT: Cumberland Ankle Instability Tool, FAAM: Foot and Ankle Ability Measure, NRS: Numeric Rating Scale, PNF: Proprioceptive Neuromuscular Facilitation, SEBT: Star Excursion Balance Test, TTB: Time-to-Boundary, WBV: Whole-Body Vibration, WBLT: Weight-Bearing Lunge Test.

**Table 2:** Quality Assessment using PEDro and RoB-2 Scales.

Author	PEDro Score (0–10)	ROB 2 Assessment
**M. Spencer Cain (2020)** [26]	07/10.	Low risk of bias across all domains
**Kyung-Min Kim (2021)** [43]	08/10.	Low risk of bias, with well-reported randomization and outcome measures
**D. Cruz-Diaz (2015)** [44]	07/10.	Some concerns regarding the blinding of participants and outcome assessors
**Mary Spencer Cain (2015)** [42]	06/10.	Low risk of bias, but some concerns in allocation concealment
**Emily A. Hal (2018)** [45]	07/10.	Some concerns due to lack of blinding in outcome assessments
**Shelley W. Linens (2016)** [46]	06/10.	Low risk of bias overall, though concerns about performance blinding
**Rafael Sierra-Guzmán (2018)** [47]	08/10.	Some concerns regarding randomization and blinding of participants
**Wen-Dien Chang (2021)** [48]	07/10.	Low risk of bias, though the randomization procedure was not fully clear
**Patrick O. Mckeon (2008)** [49]	06/10.	Some concerns due to potential bias in the measurement of outcomes
**Kyung-Min Kim (2022)** [51]	08/10.	Low risk of bias, with strong randomization and blinding practices
**Cynthia J. (2016)** [65]	07/10.	Low risk of bias, with clear blinding and allocation concealment

Legend: PEDro Score: Physiotherapy Evidence Database Score, RoB-2: Risk of Bias 2 Tool. This table assesses the quality and potential biases of each study based on the PEDro score and RoB-2 scale, indicating the overall methodological rigor and risk of bias.

## Data Availability

No data.

## References

[1] Wikstrom E.A., Hubbard-Turner T., McKeon P.O. (2013). Understanding and Treating Lateral Ankle Sprains and Their Consequences: A Constraints-Based Approach. Sports Med..

[2] McKeon P.O., Wikstrom E.A. (2016). Sensory-Targeted Ankle Rehabilitation Strategies for Chronic Ankle Instability. Med. Sci. Sports Exerc..

[3] McKeon P.O., Wikstrom E.A. (2019). The Effect of Sensory-Targeted Ankle Rehabilitation Strategies on Single-Leg Center of Pressure Elements in Those with Chronic Ankle Instability: A Randomized Clinical Trial. J. Sci. Med. Sport.

[4] Spennacchio P., Senorski E.H., Mouton C., Cabri J., Seil R., Karlsson J. (2024). A New Patient-Reported Outcome Measure for the Evaluation of Ankle Instability: Description of the Development Process and Validation Protocol. J. Orthop. Surg. Res..

[5] Hertel J., Corbett R.O. (2019). An Updated Model of Chronic Ankle Instability. J. Athl. Train..

[6] Pietrosimone B.G., McLeod M.M., Lepley A.S. (2012). A Theoretical Framework for Understanding Neuromuscular Response to Lower Extremity Joint Injury. Sports Health.

[7] Witchalls J.B., Waddington G., Adams R., Blanch P. (2014). Chronic Ankle Instability Affects Learning Rate during Repeated Proprioception Testing. Phys. Ther. Sport.

[8] Brown C., Padua D., Marshall S.W., Guskiewicz K. (2008). Individuals with Mechanical Ankle Instability Exhibit Different Motion Patterns than Those with Functional Ankle Instability and Ankle Sprain Copers. Clin. Biomech..

[9] Patti A., Gervasi M., Giustino V., Figlioli F., Canzone A., Drid P., Thomas E., Messina G., Vicari D.S.S., Palma A. (2024). The Influence of Ankle Mobility and Foot Stability on Jumping Ability and Landing Mechanics: A Cross-Sectional Study. J. Funct. Morphol. Kinesiol..

[10] Kwon Y.U. (2023). Lower Extremity Muscle Activation during the Star Excursion Balance Test in Patients with Chronic Ankle Instability and Copers. Medicina.

[11] Pijnenburg A.C.M., Bogaard K., Krips R., Marti R.K., Bossuyt P.M.M., van Dijk C.N. (2003). Operative and Functional Treatment of Rupture of the Lateral Ligament of the Ankle. A Randomised, Prospective Trial. J. Bone Jt. Surg. Br..

[12] Delahunt E., Coughlan G.F., Caulfield B., Nightingale E.J., Lin C.-W.C., Hiller C.E. (2010). Inclusion Criteria When Investigating Insufficiencies in Chronic Ankle Instability. Med. Sci. Sports Exerc..

[13] Miklovic T.M., Donovan L., Protzuk O.A., Kang M.S., Feger M.A. (2018). Acute Lateral Ankle Sprain to Chronic Ankle Instability: A Pathway of Dysfunction. Physician Sportsmed..

[14] Gribble P.A., Bleakley C.M., Caulfield B.M., Docherty C.L., Fourchet F., Fong D.T.-P., Hertel J., Hiller C.E., Kaminski T.W., McKeon P.O. (2016). Evidence Review for the 2016 International Ankle Consortium Consensus Statement on the Prevalence, Impact and Long-Term Consequences of Lateral Ankle Sprains. Br. J. Sports Med..

[15] Lin J.-Z., Hung M.-H., Ko B.-J., Lee H.-J. (2024). Analysing Lower Limb Motion and Muscle Activation in Athletes with Ankle Instability during Dual-Task Drop-Jump. Sports Biomech..

[16] Aicale R., Tarantino D., Maffulli N. (2018). Overuse Injuries in Sport: A Comprehensive Overview. J. Orthop. Surg. Res..

[17] Attenborough A.S., Hiller C.E., Smith R.M., Stuelcken M., Greene A., Sinclair P.J. (2014). Chronic Ankle Instability in Sporting Populations. Sports Med..

[18] Yeum W.-J., Lee M.-Y., Lee B.-H. (2024). The Influence of Hip-Strengthening Program on Patients with Chronic Ankle Instability. Medicina.

[19] Koshino Y., Kobayashi T. (2024). Noninstrumented Clinical Assessment of Static Postural Stability in Chronic Ankle Instability: A Systematic Review and Meta-Analysis. J. Sport Rehabil..

[20] Ortega C., Simpson J.D., Donovan L., Forsyth L., Torp D.M., Koldenhoven R.M. (2024). Gait Training Interventions for Individuals with Chronic Ankle Instability: A Systematic Review & Meta-Analysis. J. Athl. Train..

[21] Zhang L., Liu T., Zhou X., Chen J., Zhang H., Leng R., Shi H., Wang G. (2024). Gait Characteristics and Deviation Factors of Backward Walking in Patients with Chronic Ankle Instability. Sports Health.

[22] He Z., Zhu H., Ye B., Zheng Z., Liu G., Pan H., Liu R. (2024). Does Chronic Ankle Instability Patients Lead to Changes in Biomechanical Parameters Associated with Anterior Cruciate Ligament Injury during Landing? A Systematic Review and Meta-Analysis. Front. Physiol..

[23] Boccolari P., Pantaleoni F., Tedeschi R., Donati D. (2024). The Mechanics of the Collateral Ligaments in the Metacarpophalangeal Joints: A Scoping Review. Morphologie.

[24] Ricci V., Mezian K., Cocco G., Donati D., Naňka O., Farì G., Özçakar L. (2022). Anatomy and Ultrasound Imaging of the Tibial Collateral Ligament: A Narrative Review. Clin. Anat..

[25] Benedetti M.G., De Santis L., Mariani G., Donati D., Bardelli R., Perrone M., Brunelli S. (2021). Chronic Pain in Lower Limb Amputees: Is There a Correlation with the Use of Perioperative Epidural or Perineural Analgesia?. NeuroRehabilitation.

[26] Cain M.S., Ban R.J., Chen Y.-P., Geil M.D., Goerger B.M., Linens S.W. (2020). Four-Week Ankle-Rehabilitation Programs in Adolescent Athletes with Chronic Ankle Instability. J. Athl. Train..

[27] Gribble P.A. (2019). Evaluating and Differentiating Ankle Instability. J. Athl. Train..

[28] Docherty C.L., Arnold B.L., Gansneder B.M., Hurwitz S., Gieck J. (2005). Functional-Performance Deficits in Volunteers with Functional Ankle Instability. J. Athl. Train..

[29] Webster K.A., Gribble P.A. (2010). Functional Rehabilitation Interventions for Chronic Ankle Instability: A Systematic Review. J. Sport Rehabil..

[30] Halabchi F., Hassabi M. (2020). Acute Ankle Sprain in Athletes: Clinical Aspects and Algorithmic Approach. World J. Orthop..

[31] Matsui K., Burgesson B., Takao M., Stone J., Guillo S., Glazebrook M., ESSKA AFAS Ankle Instability Group (2016). Minimally Invasive Surgical Treatment for Chronic Ankle Instability: A Systematic Review. Knee Surg. Sports Traumatol. Arthrosc..

[32] Alexandre É., Monteiro D., SottoMayor R., Jacinto M., Silva F.M., Cid L., Duarte-Mendes P. (2024). Assessing Functional Ankle Instability in Sport: A Critical Review and Bibliometric Analysis. Healthcare.

[33] Park H.S., Oh J.K., Kim J.Y., Yoon J.H. (2024). The Effect of Strength and Balance Training on Kinesiophobia, Ankle Instability, Function, and Performance in Elite Adolescent Soccer Players with Functional Ankle Instability: A Prospective Cluster Randomized Controlled Trial. J. Sports Sci. Med..

[34] Debolt L., Hamon J., Hu J., Vickers T., Hung Y.-J. (2024). Effects of Ankle Compression Garments on Fatigue and Single-Leg Balance in Collegiate Basketball Players. Int. J. Exerc. Sci..

[35] Lanfranchi E., Vandelli S., Boccolari P., Donati D., Platano D., Tedeschi R. (2024). Efficacy and Patient Acceptability of 3 Orthosis Models for Radial Nerve Palsy. Hand Surg. Rehabil..

[36] Donati D., Vita F., Tedeschi R., Galletti S., Biglia A., Gistri T., Arcuri P., Origlio F., Castagnini F., Faldini C. (2023). Ultrasound-Guided Infiltrative Treatment Associated with Early Rehabilitation in Adhesive Capsulitis Developed in Post-COVID-19 Syndrome. Medicina.

[37] Liu S., Tang J., Hu G., Xiong Y., Ji W., Xu D. (2024). Blood Flow Restriction Training Improves the Efficacy of Routine Intervention in Patients with Chronic Ankle Instability. Sports Med. Health Sci..

[38] Chang S., Tan Y., Cheng L., Zhou L., Wang B., Liu H. (2024). Effect of Strength Training with Additional Acupuncture on Balance, Ankle Sensation, and Isokinetic Muscle Strength in Chronic Ankle Instability among College Students. Front. Physiol..

[39] Giannini S., Ruffilli A., Pagliazzi G., Mazzotti A., Evangelisti G., Buda R., Faldini C. (2014). Treatment Algorithm for Chronic Lateral Ankle Instability. Muscles Ligaments Tendons J..

[40] Petersen W., Rembitzki I.V., Koppenburg A.G., Ellermann A., Liebau C., Brüggemann G.P., Best R. (2013). Treatment of Acute Ankle Ligament Injuries: A Systematic Review. Arch. Orthop. Trauma Surg..

[41] Ec R.-M. (2012). Chronic Ankle Instability: Diagnosis and Treatment. Arch. Orthop. Trauma Surg..

[42] Ms C., Sw G., Sw L. (2017). Effects of a 4-Week Biomechanical Ankle Platform System Protocol on Balance in High School Athletes With Chronic Ankle Instability. J. Sport Rehabil..

[43] Kim K.-M., Estudillo-Martínez M.D., Castellote-Caballero Y., Estepa-Gallego A., Cruz-Díaz D. (2021). Short-Term Effects of Balance Training with Stroboscopic Vision for Patients with Chronic Ankle Instability: A Single-Blinded Randomized Controlled Trial. Int. J. Environ. Res. Public Health.

[44] Cruz-Diaz D., Lomas-Vega R., Osuna-Pérez M.C., Contreras F.H., Martínez-Amat A. (2015). Effects of 6 Weeks of Balance Training on Chronic Ankle Instability in Athletes: A Randomized Controlled Trial. Int. J. Sports Med..

[45] Hall E.A., Chomistek A.K., Kingma J.J., Docherty C.L. (2018). Balance- and Strength-Training Protocols to Improve Chronic Ankle Instability Deficits, Part I: Assessing Clinical Outcome Measures. J. Athl. Train..

[46] Linens S.W., Ross S.E., Arnold B.L. (2016). Wobble Board Rehabilitation for Improving Balance in Ankles with Chronic Instability. Clin. J. Sport Med..

[47] Sierra-Guzmán R., Jiménez-Diaz F., Ramírez C., Esteban P., Abián-Vicén J. (2018). Whole-Body-Vibration Training and Balance in Recreational Athletes with Chronic Ankle Instability. J. Athl. Train..

[48] Chang W.-D., Chen S., Tsou Y.-A. (2021). Effects of Whole-Body Vibration and Balance Training on Female Athletes with Chronic Ankle Instability. J. Clin. Med..

[49] McKeon P.O., Ingersoll C.D., Kerrigan D.C., Saliba E., Bennett B.C., Hertel J. (2008). Balance Training Improves Function and Postural Control in Those with Chronic Ankle Instability. Med. Sci. Sports Exerc..

[50] Plisky P.J., Gorman P.P., Butler R.J., Kiesel K.B., Underwood F.B., Elkins B. (2009). The Reliability of an Instrumented Device for Measuring Components of the Star Excursion Balance Test. N. Am. J. Sports Phys. Ther..

[51] Kim K.-M., Estepa-Gallego A., Estudillo-Martínez M.D., Castellote-Caballero Y., Cruz-Díaz D. (2022). Comparative Effects of Neuromuscular- and Strength-Training Protocols on Pathomechanical, Sensory-Perceptual, and Motor-Behavioral Impairments in Patients with Chronic Ankle Instability: Randomized Controlled Trial. Healthcare.

[52] Zhao Q., Zhang Z., Gu X. (2024). A Matching and Localization Study of Iliac Bone Graft for Repair of Talar Cartilage Injury Secondary to Lateral Ankle Instability. J. Orthop. Surg..

[53] Sonobe T., Watanabe K., Endo Y., Nikaido T., Matsumoto Y. (2024). A Professional Basketball Player Who Suffered an Open Ankle Dislocation Without an Associated Fracture Achieves His Prior Performance Level Three Months Later. Cureus.

[54] Zhou Z., Zhou H., Jie T., Xu D., Teo E.-C., Wang M., Gu Y. (2024). Analysis of Stress Response Distribution in Patients with Lateral Ankle Ligament Injuries: A Study of Neural Control Strategies Utilizing Predictive Computing Models. Front. Physiol..

[55] Labanca L., Tedeschi R., Mosca M., Benedetti M.G. (2024). Individuals with Chronic Ankle Instability Show Abnormalities in Maximal and Submaximal Isometric Strength of the Knee Extensor and Flexor Muscles. Am. J. Sports Med..

[56] Tedeschi R. (2024). Unlocking the Power of Motor Imagery: A Comprehensive Review on Its Application in Alleviating Foot Pain. Acta Neurol. Belg..

[57] Anderson D.D., Wilken J., Ledoux W., Lenz A.L., Easley M.E., de Cesar Netto C. (2024). Ankle Osteoarthritis: Toward New Understanding and Opportunities for Prevention and Intervention. J. Orthop. Res..

[58] Goetz J., Baier C., Vitzethum G., Grifka J., Maderbacher G., Springorum H.-R. (2024). Postural Stability after Operative Reconstruction of the AFTL in Chronic Ankle Instability Comparing Three Different Surgical Techniques. Open Med. Wars.

[59] Choi J.Y., Suh J.S., Park J.H., Asfaw T.T. (2024). High Incidence of Post-Operative Re-Sprain Following Suture Tape Implantation for Anterior Talofibular Ligament Insufficiency and Risk Factors for Post-Operative Re-Sprain. Knee Surg. Sports Traumatol. Arthrosc..

[60] Tedeschi R. (2024). Exploring the Potential of iPhone Applications in Podiatry: A Comprehensive Review. Egypt. Rheumatol. Rehabil..

[61] Tedeschi R. (2023). What Are the Benefits of Five-Toed Socks? A Scoping Review. Reabil. Moksl. Slauga Kineziter. Ergoter..

[62] Peters: Joanna Briggs Institute Reviewer’s Manual, JBI—Google Scholar. https://scholar-google-com.ezproxy.unibo.it/scholar_lookup?hl=en&publication_year=2020&author=MDJ+Peters&author=C+Godfrey&author=P+McInerney&author=Z+Munn&author=AC+Tricco&author=H+Khalil&title=Joanna+Briggs+Institute+Reviewer%27s+Manual%2C+JBI.

[63] Tricco A.C., Lillie E., Zarin W., O’Brien K.K., Colquhoun H., Levac D., Moher D., Peters M.D.J., Horsley T., Weeks L. (2018). PRISMA Extension for Scoping Reviews (PRISMA-ScR): Checklist and Explanation. Ann. Intern. Med..

[64] Page M.J., McKenzie J.E., Bossuyt P.M., Boutron I., Hoffmann T.C., Mulrow C.D., Shamseer L., Tetzlaff J.M., Akl E.A., Brennan S.E. (2021). The PRISMA 2020 Statement: An Updated Guideline for Reporting Systematic Reviews. BMJ.

[65] Wright C.J., Linens S.W., Cain M.S. (2017). A Randomized Controlled Trial Comparing Rehabilitation Efficacy in Chronic Ankle Instability. J. Sport Rehabil..

[66] Taube W., Gruber M., Gollhofer A. (2008). Spinal and Supraspinal Adaptations Associated with Balance Training and Their Functional Relevance. Acta Physiol..

[67] Al B., Mg B., Pm N. (2006). Neuroplasticity after Spinal Cord Injury and Training: An Emerging Paradigm Shift in Rehabilitation and Walking Recovery. Phys. Ther..

[68] Seidler R.D., Bernard J.A., Burutolu T.B., Fling B.W., Gordon M.T., Gwin J.T., Kwak Y., Lipps D.B. (2010). Motor Control and Aging: Links to Age-Related Brain Structural, Functional, and Biochemical Effects. Neurosci. Biobehav. Rev..

[69] Priplata A.A., Patritti B.L., Niemi J.B., Hughes R., Gravelle D.C., Lipsitz L.A., Veves A., Stein J., Bonato P., Collins J.J. (2006). Noise-Enhanced Balance Control in Patients with Diabetes and Patients with Stroke. Ann. Neurol..

[70] Balter S.G., Stokroos R.J., Akkermans E., Kingma H. (2004). Habituation to Galvanic Vestibular Stimulation for Analysis of Postural Control Abilities in Gymnasts. Neurosci. Lett..

[71] Ritzmann R., Kramer A., Gruber M., Gollhofer A., Taube W. (2010). EMG Activity during Whole Body Vibration: Motion Artifacts or Stretch Reflexes?. Eur. J. Appl. Physiol..

[72] Hodges P.W., Tucker K. (2011). Moving Differently in Pain: A New Theory to Explain the Adaptation to Pain. Pain.

[73] Cram J.R., Kasman G.S., Holtz J. (1998). Introduction to Surface Electromyography.

